# Green synthesis of bimetallic Ag/ZnO@Biohar nanocomposite for photocatalytic degradation of tetracycline, antibacterial and antioxidant activities

**DOI:** 10.1038/s41598-022-11014-0

**Published:** 2022-05-05

**Authors:** Mohamed Hosny, Manal Fawzy, Abdelazeem S. Eltaweil

**Affiliations:** 1 Green Technology Group, Environmental Sciences Department, Faculty of Science, Alexandria University, Alexandria, 21511 Egypt; 2 National Egyptian Biotechnology Experts Network, National Egyptian Academy for Scientific Research and Technology, Cairo, Egypt; 3 Department of Chemistry, Faculty of Science, Alexandria University, Alexandria, 21321 Egypt

**Keywords:** Environmental sciences, Nanoscience and technology

## Abstract

In this work, a simple and green synthesis procedure for phytofabrication Zinc oxide-silver supported biochar nanocomposite (Ag/ZnO@BC) via* Persicaria salicifolia* biomass is investigated for the first time to uphold numerous green chemistry such as less hazardous chemical syntheses. XRD technique showed the crystal structure of the phytosynthesized Ag/ZnO@BC, whereas UV–visible spectroscopy, FT-IR, SEM, EDX, TEM, and XPS analyses indicated the successful biosynthesis of the nanocomposite. Testing the photocatalytic potential of this novel nanocomposite in the removal of TC under different conditions unraveled its powerful photodegradation efficiency that reached 70.3% under the optimum reaction conditions: TC concentration; 50 ppm, pH; 6, a dose of Ag/ZnO@BC; 0.01 g, temperature; 25 °C, and H_2_O_2_ concentration; 100 mM. The reusability of Ag/ZnO@BC was evident as it reached 53% after six cycles of regeneration. Ag/ZnO@BC was also shown to be a potent antimicrobial agent against *Klebsiella pneumonia* as well as a promising antioxidant material. Therefore, the current work presented a novel nanocomposite that could be efficiently employed in various environmental and medical applications.

## Introduction

Wastewater recycling, for different purposes, needs financially-feasible and effective technologies for removing toxic organic and inorganic pollutants^1–3^. Such an approach has been manifested as an effective option for the management of wastewater in view of the emerging green technologies^4,5^. This trend has become a priority in most of the world’s countries stemming from the water need for an available supply that could solve the problem of water shortage caused by the fluctuation in precipitation and the irregular availability of other water sources, especially for agriculture^6^. Water reserves are rapidly depleting as a result of the exponential growth of the world's population and global warming^7^.

Biochar (BC) is a carbon-rich solid material that is formed through pyrolysis of biomass at high temperatures in an oxygen-free (or low-oxygen) condition. Outstanding properties including biocompatibility, facile synthesis, easy regeneration, and surface modification have rendered the biochar to be harnessed in lots of different applications. Biochar has been comprehensively examined for the removal of toxic pollutants, where it is utilized to immobilize toxic materials including heavy metals and organic contaminants by adsorption and also as a support for various types of catalysts that could be used for degrading perilous contaminants in advanced oxidation processes (AOPs)^8^. However, there are some negative aspects when using pristine biochar, such as limited porosity and functional units. Subsequently, various amendments including chemical, physical, and magnetic modification as well as the impregnation with nanomaterials especially the bimetallic nanoparticles have been employed to improve biochar efficiency. The monometallic nanoparticle systems have several drawbacks, including limited pH range of action, and poor recyclability^9^. On the other hand, bimetallic nanoparticles, which are made up of two metals, have been concluded to be efficient in resolving these negative points and thereby enhancing the performance of these nanomaterials by virtue of their nanocomponents properties and also the new properties that emerge as a result of the synergic effect of bimetallic nanoparticles^10^.

Among the numerous types of nanoparticles, gold and silver garnered most of the researchers' consideration owing to their outstanding characteristics and applicability in various fields including antibacterial, antioxidant, photocatalytic, and other applications^11,12^. Also, zinc oxide (ZnO) nanoparticles have gotten a lot of interest because of their numerous applications including UV light-emitting diodes and catalysts^13^. The nanoparticle synthesis employing green chemistry techniques has several positive aspects compared to the conventional physical and chemical routes, mainly being easy to synthesize, does not contaminate the environment, and relatively inexpensive^14,15^. Thus, obviating the usage of toxic chemicals and the high cost of production that is frequently attributed to physical and chemical synthesis techniques^16,17^.

Tetracyclines, which are broad-spectrum antibiotics for humans and animals that are synthesized by modifying natural tetracycline to form several new compounds^18^, constitute another perilous source of toxic organic pollutants that severely affect water quality. Tetracycline (TC) has been considered as one of the highly popular drugs particularly in the last two years because of its utilization as an antibacterial as well as an antiviral against the progression of Coronavirus. These tetracyclines, on the other hand, are challenging to get decomposed, and only a limited percentage of them can be absorbed by human or animals' bodies^19^ causing a variety of environmental and health problems^20,21^. Accordingly, a suitable and cost-effective treatment strategy is highly required.

Based on the available literature, this is the first study for using the extract of *Persicaria salicifolia* (Brouss. ex Willd.) Assenov., which is a common hydrophyte species that found alongside the Nile Delta region in Egypt^22^, in the synthesis of AgNPs, ZnONPs, and also its biomass for the preparation of biochar material Ag/ZnO@BC nanocomposite in a facile, cost-effective, and complete green synthesis procedure, which represents the main novelty of this work. Subsequently, the target of this work is threefold: (1) to examine the synergistic effect of green synthesized AgNPs and ZnONPs supported on *P. salicifolia* biochar in the photocatalytic degradation of perilous organic pollutants such as Tetracycline (TC) under different reaction conditions, for the sake of optimizing the process, (2) to examine the antibacterial efficiency of the prepared nanocomposite, (3) to test its antioxidant potentiality.

## Materials and methods

### Materials

Zinc nitrate hexahydrate Zn(NO_3_)_2_.6H_2_O and silver nitrate (99.9%, AgNO_3_) were purchased from Merck, USA. Tetracycline was purchased from EL NASR pharmaceutical company, Egypt.

### Preparation of pristine biochar

*Persicaria salicifolia* (*P. salicifolia*) was collected from the northern coast of Egypt, specifically from Kafr El-Dawar governorate. Plant material was collected in accordance with applicable national and international guidelines^23^. Permission for collecting the investigated plant species for scientific purposes was obtained from Environmental Sciences Department, Alexandria University. Plant specimens were identified by Professor Manal Fawzy according to Boulos^24^. Voucher specimens were deposited in Tanta University Herbarium (TANE) with voucher Numbers: 14122–14127, which is a public herbarium providing access to the deposited material. The plant stem organ was separated from the whole plant and then the stem was cleaned many times with deionized water (D.W.) to remove any debris. Subsequently, it was fractured and dried in air before starting the overnight oven drying at 60 °C. Dry stems were then grinded into a fine powder using a mixer. Moreover, ten grams of the fine powder was pyrolyzed for 3 h at 550 °C in a muffle furnace to get immaculate biochar.

### Preparation of Ag/ZnO@BC

**Figure 1:**
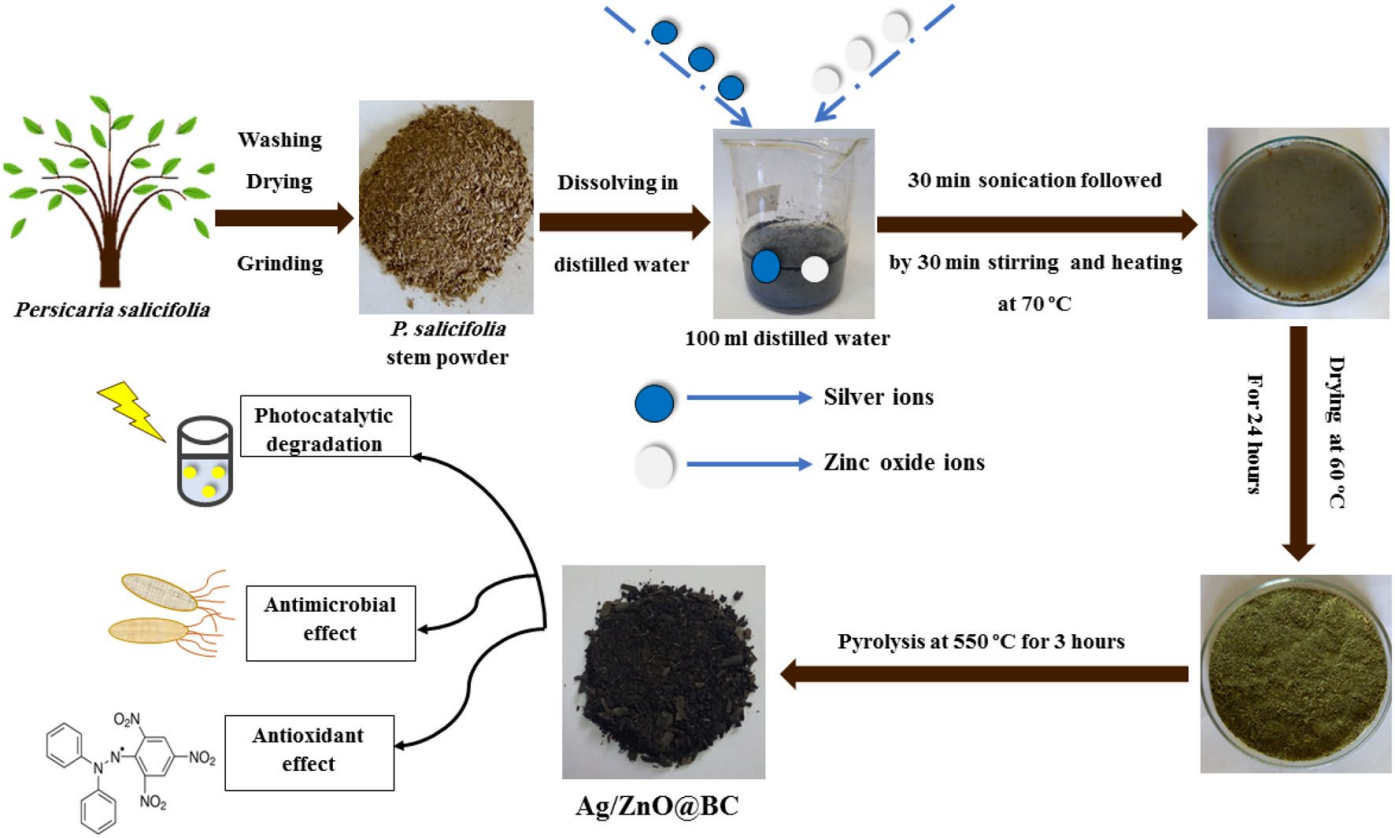
Schematic illustration of the green synthesis of Ag/ZnO@BC.

4.9 g of *P. salicifolia* powder were mixed with 0.175 g of Zn(NO_3_)_2_.6H_2_O and 0.08 g of AgNO_3_ with a wt% ratio of 1:1 for Zn : Ag and a wt% ratio of 2:100 for both Ag: biochar and Zn : biochar. Subsequently, this mixture was dissolved in 100 mL D.W and sonicated for about 30 min followed by stirring for another 30 min accompanied by heating at 70 °C. Furthermore, this mixture was oven-dried at 60 °C for a period of 24 h. After that, it was pyrolyzed using a muffle furnace at 550 °C for 3 h to get both ZnO and Ag ions reduced into ZnONPs and AgNPs on the surface of the *P. salicifolia* dried powder which will be pyrolyzed in a limited amount of oxygen to obtain the biochar material in order to finally obtain the novel biochar nanocomposite (Ag/ZnO@BC). The synthesis process is displayed in Fig. 1.

### Characterization of Ag/ZnO@BC

The phytosynthesis of Ag/ZnO@BC was indicated by UV–visible spectroscopy (Genesis 10S UV–VIS spectrophotometer, Thermo scientific). Crystallinity of pristine biochar and Ag/ZnO@BC were determined by XRD (Siemens D-5000) with Cu Kα radiation (λ = 0.154 nm). SEM (model EVO 40, Zeiss) attached to EDX (model Bruker EDX system) were used for the examination of the morphology and analysis of elements. TEM measurements were conducted on a JEOL model 1200EX instrument operated at an accelerating voltage of (80 kV). XPS analysis was collected ESCALAB250Xi (Thermo Scientific, China) spectrometer with a monochromatic Al Kα radiation source (energy 1486.68 eV) at 164 W. FT-IR spectrums were attained over the range of 4000–400 cm^−1^ using a TENSOR-5, Bruker FTIR Spectrometer. TGA analysis was carried out using Pyris-1, Diamond TG/DTA PerkinElmer. Zeta potentials of synthesized samples were determined using ZetaPALS, Brookhaven, USA to measure their surface charge and stability.

### Photocatalytic experiments

Photocatalytic activity of the green synthesized Ag/ZnO@BC against TC was evaluated. The effect of different doses of Ag/ZnO@BC (0.005, 0.0075, and 0.01 g) was tested with 20 mL of TC solutions. As well as the effect of the initial concentration of TC (25, 50, and 75 ppm) was investigated. Four different levels of pH (2, 4, 6, and 8) were tested. Three different temperatures were used including 10 °C, 25 °C, and 40 °C. In addition, the effect of free radicals such as H_2_O_2_ was examined utilizing various concentrations including 25, 50, 75, and 100 mM. All these factors were studied to search for the optimum conditions for TC removal. Control experiments were carried out using the pristine biochar with the same experimental conditions. Test and control solutions were mixed for 30 min in dark conditions for the sake of adsorption/desorption equilibration to eliminate the adsorption effect and determine the actual photocatalytic degradation efficiency. Then, the solutions were subjected to stirring under a xenon lamp as a UV light source (λ ≈ 320 nm and the intensity ≈ 500 watts) and monitored. Next, 2 mL aliquots were taken and centrifuged at 17,000 rpm for 1 min to separate the solid nanocatalyst, then diluted with D.W. to 4 mL to be within the absorbance range of the UV–visible system. The absorbance of the resultant diluted supernatant of control and test solutions was measured at 360 nm in a quartz cuvette (path length 1 cm) using Genesis 10S UV–VIS spectrophotometer, Thermo scientific. Percentages of TC photodegradation were measured by the following formula:

1 $$ \% {\text{Degradation}} = {1}00 \times ({\text{A}}_{0} - {\text{A}}){\text{/A}}_{0} $$

where (A_0_) denotes the initial absorbance, while (A) indicates the final absorbance.

### Antimicrobial test

#### Inoculum preparation

*Bacillus subtilis* (ATCC 6633), *Staphyllococcus aureus* (ATCC 25923), *Escherichia coli* (ATCC 8739), and *Klebsiella pneumonia* (ATCC 1388) were used. The colonies' turbidity was compared to a 0.5 McFarland turbidity standard, which is equivalent to 2 × 10^8^ CFU/mL.

#### Preparation of seeded agar

Muller Hinton agar is dissolved in D.W and sterilized in the autoclave after being distributed into 25 mL portions into 6 separate flasks. Flasks were shaken then decanted onto sterile petri dishes and allowed to solidify.

#### Placing of tested materials (Ag/ZnO@BC)

Ag/ZnO@BC was placed in wells after filtration for the sake of sterilization. Subsequently, the plates were put in the refrigerator overnight to allow diffusion of Ag/ZnO@BC.

#### Incubation

Plates were left at 35 ± 2 °C for duration of 24 h.

#### Reading results

All measurements were taken with the naked eye while seeing the back of the Petri dish a few inches above a black background that was lit by reflected light.

### Antioxidant activity of Ag/ZnO@BC (DPPH assay)

The antioxidant efficiency of Ag/ZnO@BC was determined using the DPPH (2, 2-diphenyl-1-picrylhydrazyl) test to measure free radical scavenging activity. Triplicates of the assay were performed. 1 mL of Ag/ZnO@BC liquid sample was mixed with 1 mL of DPPH (0.2 mM) and control experiment of DPPH that did not include nanocomposites during the process. These combinations were mixed for 3 min at room temperature in the dark. The concentration of radical is then determined by measuring the reduction in absorbance percentage of the mixture after 20 min. The control had been set to over. At 517 nm, the change in absorbance was estimated. As a positive control, vitamin C (ascorbic acid) was employed. The following equation was used to calculate the radical scavenging activity.

2 $$ {\text{Radical \,scavenging \,activity}} = \frac{{(Control \,absorbance - Sample \,absorbance) \times 100}}{{Control \,absorbance}} $$

The absorbance in the absence of antioxidants is called control absorbance, whereas the absorbance in the presence of antioxidants as Ag/ZnO@BC or Vitamin C is called sample absorbance.

### Statistical analysis

The experimental work was done in triplicate (n = 3), and the results were given as a mean value with the standard deviation (± SD) subtracted.

## Results and discussion

### UV–visible spectroscopy

**Figure 2:**
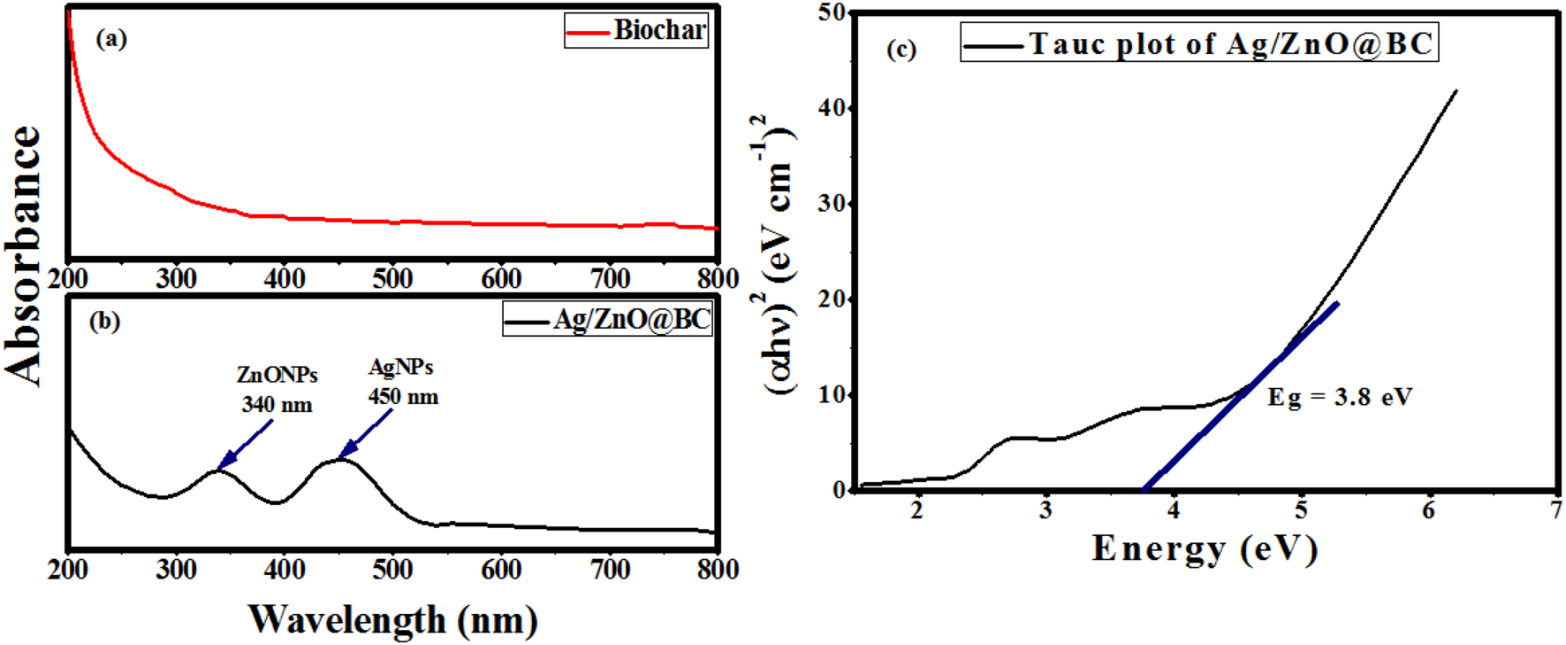
UV–visible spectra of Biochar (**a**) and Ag/ZnO@BC (**b**) Tauc plot of ZnO-Ag- biochar (**c**).

The excitation created by a light source at a specific wavelength causes a unique peak at that wavelength called surface plasmonic resonance (SPR) in UV–visible spectroscopy. The morphology of nanoparticles often determines the shape and location of the SPR peak. Regarding the UV–visible range of the pristine biochar (Fig. 2a), no bands were detected. However, two SPR bands were observed in Fig. 2b at 340 nm and 450 nm which are characteristic for ZnONPs and AgNPs, respectively, indicating the reduction of both zinc oxide and silver ions on the biochar surface and the phytoformation of Ag/ZnO@BC nanocomposite. The observed SPR band of AgNPs was concomitant with other results that aimed at the synthesis of AgNPs nanocomposites. Moreover, the obtained results in the current study are similar to those previously reported that ZnONPs exhibit a characteristic SPR band ranging from 330 to 380 nm^25^. Furthermore, it has been noticed that the bandgap energy (Eg) of Ag/ZnO@BC was calculated by the Tauc plot and it was found to be 3.8 eV as shown in Fig. 2c. Such a narrow bandgap was resulting from the introduction of ZnO and Ag nanoparticles into the nanocomposite which is in agreement with Gurgur et al.^26^ who reported a bandgap energy of 3.24 eV for ZnONPs that was narrowed to 3.12 eV after modifying with AgNPs using *Bridelia ferruginea* as well as Rajendran et al.^27^ who did find a similar result when the bandgap energy of ZnO was diminished from 3.28 to 3.12 eV after doping it with AgNPs. Also, the current result was in line with Cheraghcheshm et al.^28^ who reported a bandgap energy of 3.22 eV for ZnO-Ag nanocomposite. Therefore, the deposition of ZnONPs and AgNPs on the biochar surface diminishes the bandgap energy of the pristine biochar and facilitates the creation of new energy states in Ag/ZnO@BC caused by Ag-C bonds formed as a result of AgNPs association with biochar's carbon content^29^. Thus, Ag/ZnO@BC could be harnessed efficiently in the photocatalytic degradation of toxic pollutants.

### FT-IR spectroscopy

**Figure 3:**
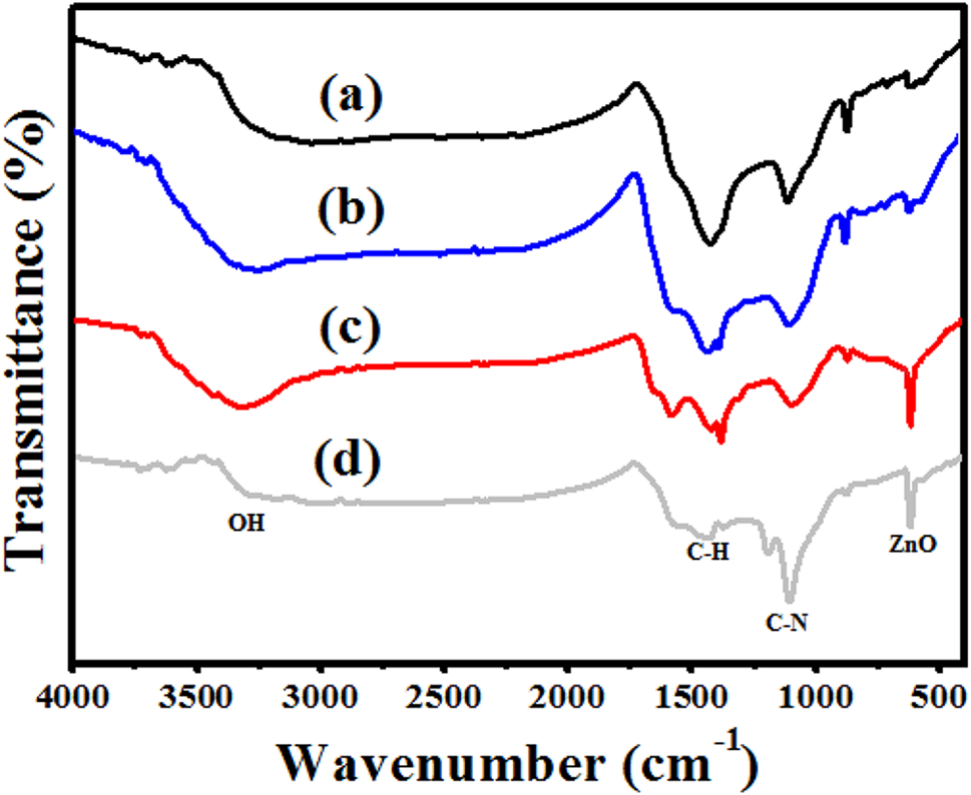
FT-IR spectroscopy of Biochar (**a**), Ag/biochar (**b**) ZnO/biochar (**c**), and Ag/ZnO@BC (**d**).

FTIR spectroscopy is frequently utilized to recognize functional groups that may contribute to the reduction and stabilization of phytosynthesized nanoparticles^30,31^. Regarding the FTIR spectrum of the pristine biochar (Fig. 3a) three functional groups were observed including a stretching vibration of hydrogen-bonded O–H group at approximately 3330 cm^−1^, C–H bending at around 1430 cm^−1^, and C–N group at 1116 cm^−1^, which are all related to biomolecules of *P. salicifolia*^4^. These peaks were also detected in the spectrum of both Ag/biochar and ZnO/biochar (Fig. 3b,c) yet with different intensity, indicating their participation in reducing both AgNPs and ZnONPs on the biochar surface. In addition, a new ZnO peak was detected at 617 cm^−1^ in ZnO/biochar confirming the formation of ZnO nanoparticles^32^. All these functional groups appeared in the spectrum of Ag/ZnO@BC but with different intensities due to the interaction among Ag, ZnO, and the biochar as shown in Fig. 3d and these results were similar to Sajjad et al.^33^ who mentioned a comparable result when doping Cu with ZnO using the extract of *Euphorbia milii*. Accordingly, it was concluded that the phytoconstituents of *P. salicifolia* present in its biochar such as flavonoids, terpenoids, alkaloids, and glycosides^34^ were responsible for the reduction and stabilization of ZnONPs and AgNPs on the surface of biochar. Therefore, this result confirmed the successful formation of Ag/ZnO@BC nanocomposite.

### Zeta potential

**Figure 4:**
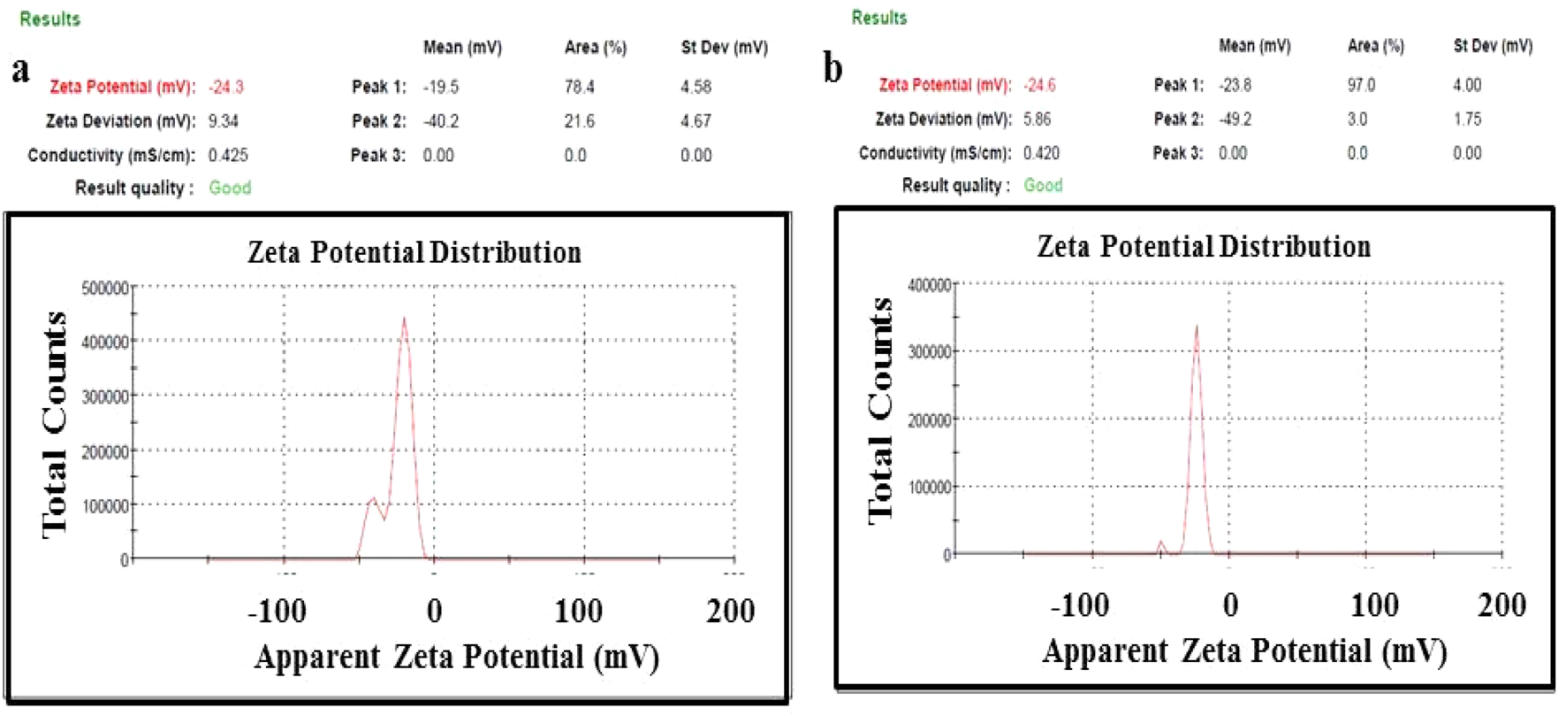
Zeta potential of biochar (**a**) and Ag/ZnO@BC composite (**b**).

Zeta potential is a useful surface characteristic for determining the stability and surface charge of colloidal materials^35,36^. Potential measurement can reflect charge alternation on materials surfaces. The sample concentration utilized for measurement is often at nanomolar concentration, meaning that it is a particularly sensitive approach for nanomaterial investigation^37^. In the current study, the zeta potential of the pristine biochar was − 24.3 mV (Fig. 4a) indicating the presence of a high concentration of bioactive ingredients in *P. salicifolia* pristine biochar material yet it changed to − 24.6 (Fig. 4b) after the formation of Ag/ZnO@BC nanocomposite confirming the successful deposition of ZnO and Ag nanoparticles on the biochar surface. The stability of Ag/ZnO@BC owing to the repulsion forces between its negatively charged particles could be concluded by the detected negative zeta potential^38^, which is similar to that obtained by Hassan et al.^39^ who detected a negative value (− 25.6 mV) for biochar nanocomposite and in line with Dheyab et al.^40^ who did enhance the zeta potential value from − 31.3 mV for Fe_3_O_4_ nanoparticles to − 45.3 mV after coating these nanoparticles with citric acid.

### SEM and EDX analyses

**Figure 5:**
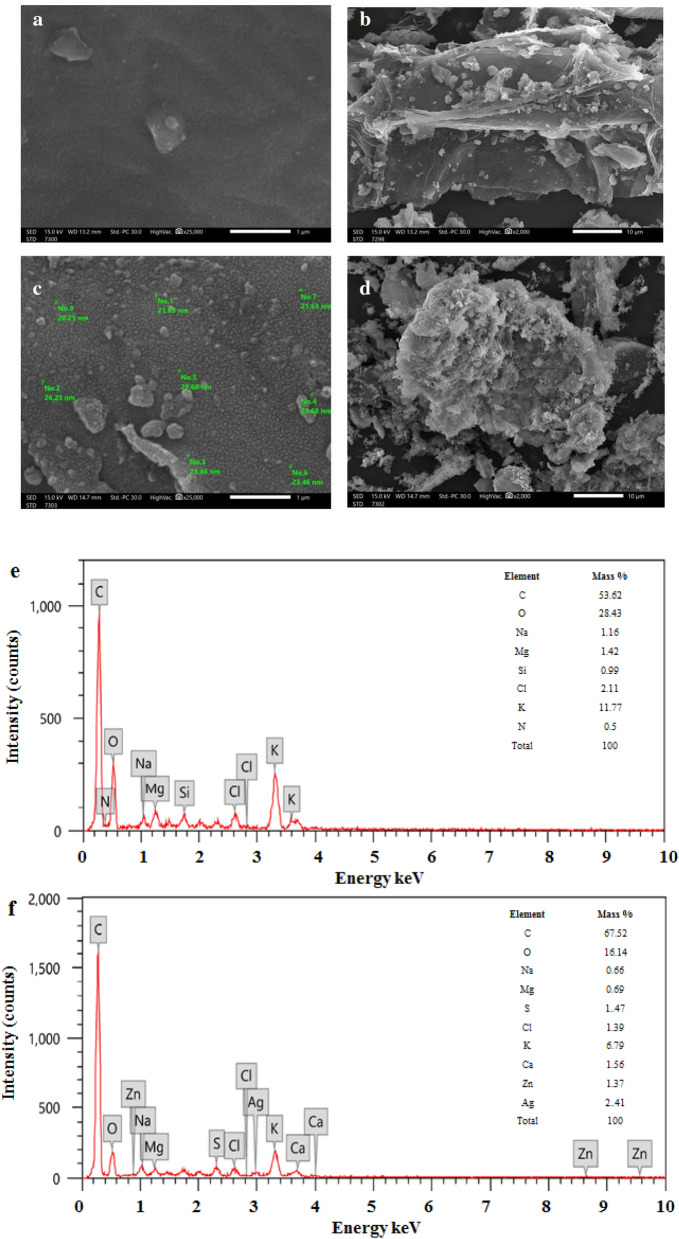
SEM images of biochar (**a**) and (**b**), Ag/ZnO@BC (**c**) and (**d**), EDX spectrum of pristine biochar (**e**), and Ag/ZnO@BC (**f**).

As it was previously employed by many workers, SEM analysis was utilized to investigate morphological surface features of biochar before and after modification with ZnONPs and AgNPs and to inspect porosity, size, and shape of ZnONPs and AgNPs supported on the biochar surface. Using SEM only helps to provide data about surface structure but together with EDX, it can be used for the identification and quantification of attached particles^41^. The pristine biochar and Ag/ZnO@BC nanocomposite were illustrated in Fig. 5a, b and Fig. 5c, d respectively. SEM results unraveled a porous structure in both materials in this work as the release of materials in the form of tiny volatile molecules such as CO, CO_2_, CH_4_, and H_2_O during the heat conversion process (pyrolysis) is widely thought to cause porosity^42^. The abundant distribution of white particles on the surface of Ag/ZnO@BC is quite apparent in Fig. 5c, d, which were not existing in the biochar (Fig. 5a, b), denoting the successful phytosynthesis of ZnONPs and AgNPs on biochar’s surface. Various elements were detected in the EDX spectrum of the pristine biochar including C and O as displayed in Fig. 5e that are considered as the major elements in most biochar materials, particularly the carbon as it increases with increasing pyrolysis temperature^43,44^. In addition, other elements were observed such as Na, Mg, Si, Cl, K, and N with different percentages, which are considered as the primary plant ingredients^45^. Almost most of these elements were detected in the EDX spectrum of Ag/ZnO@BC as shown in Fig. 5f. Additionally, the detection of clear signals for nano silver at 3 keV in the current study was in line with recent results which were reported by others such as Ma et al.^46^ who phytosynthesized AgNPs using soybean. In addition, signals that are attributed to Zn were observed at 0.9, 8.65, and 9.6 keV in the same figure, and they were found to be concomitant with Shaban et al.^47^ who modified cotton fibers with ZnONPs. Thus, indicating the synthesis of Ag/ZnO@BC. The Elemental analysis of Ag/ZnO@BC’s surface (inset Fig. 5f) showed that the zero-valent Ag percentage was 2.41% while the elemental Zn percentage was 1.37% which are close to the percentages of both Zinc oxide and silver ions (2%) which were originally used on the biochar surface confirming the high efficiency of the extract of *P. salicifolia* in reducing ions of zinc and silver on the biochar’s surface. Moreover, the particle size of the dispersed nanoparticles on the surface of the biochar was observed ranging from 20 to 30 nm which was similar to other research works^48^.

### TEM analysis

**Figure 6:**
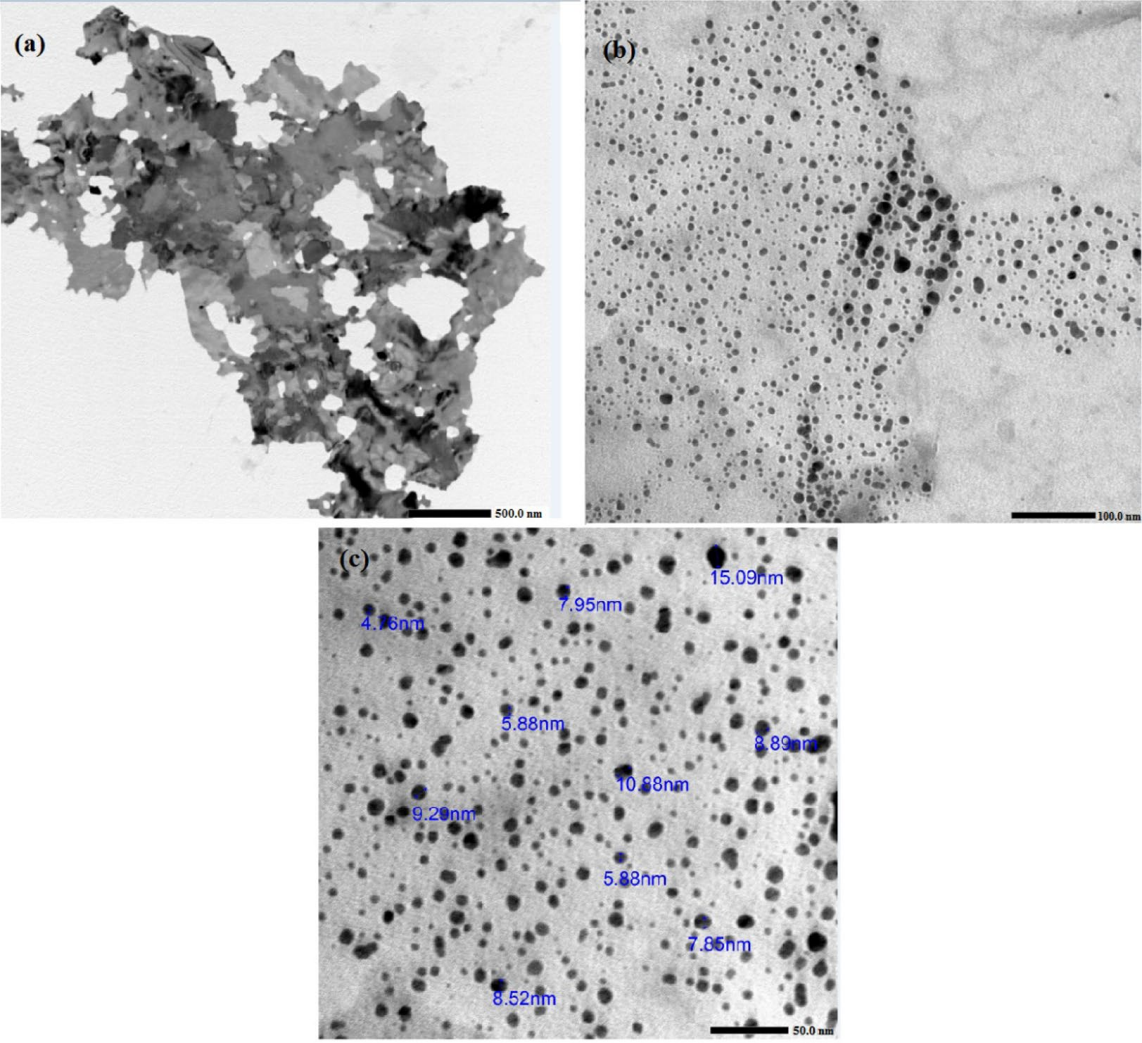
TEM images of Ag/ZnO@BC at 500 nm (**a**), 100 nm (**b**), and 50 nm (**c**)**.**

TEM analysis which is commonly utilized in determining the morphology of synthesized nanoparticles^49,50^, was used in this study to examine the size and shape of the phytosynthesized AgNPs and ZnONPs on the biochar surface. TEM images showed the distribution of AgNPs and ZnONPs on biochar surface as shown in Fig. 6a. On a higher scale, AgNPs and ZnONPs were shown to be spherical with various particle sizes up to 20 nm as displayed in Fig. 6b, c is in agreement with the size range obtained by SEM analysis confirming the successful green synthesis of Ag/ZnO@BC and indicating its potential applicability in different applications as a result of this small nanoparticle size. The obtained result is in agreement with the results obtained by Ravikumar et al.^51^ as they observed similar shapes for the nanocomposite Ag-TiO_2_@Pd/C with sizes ranging from 10 to 40 nm as well as Zhang et al.^52^ who detected a particle size of around 20 nm for γ-Fe_2_O_3_-ZnO-biochar nanocomposite.

### XPS analysis

**Figure 7:**
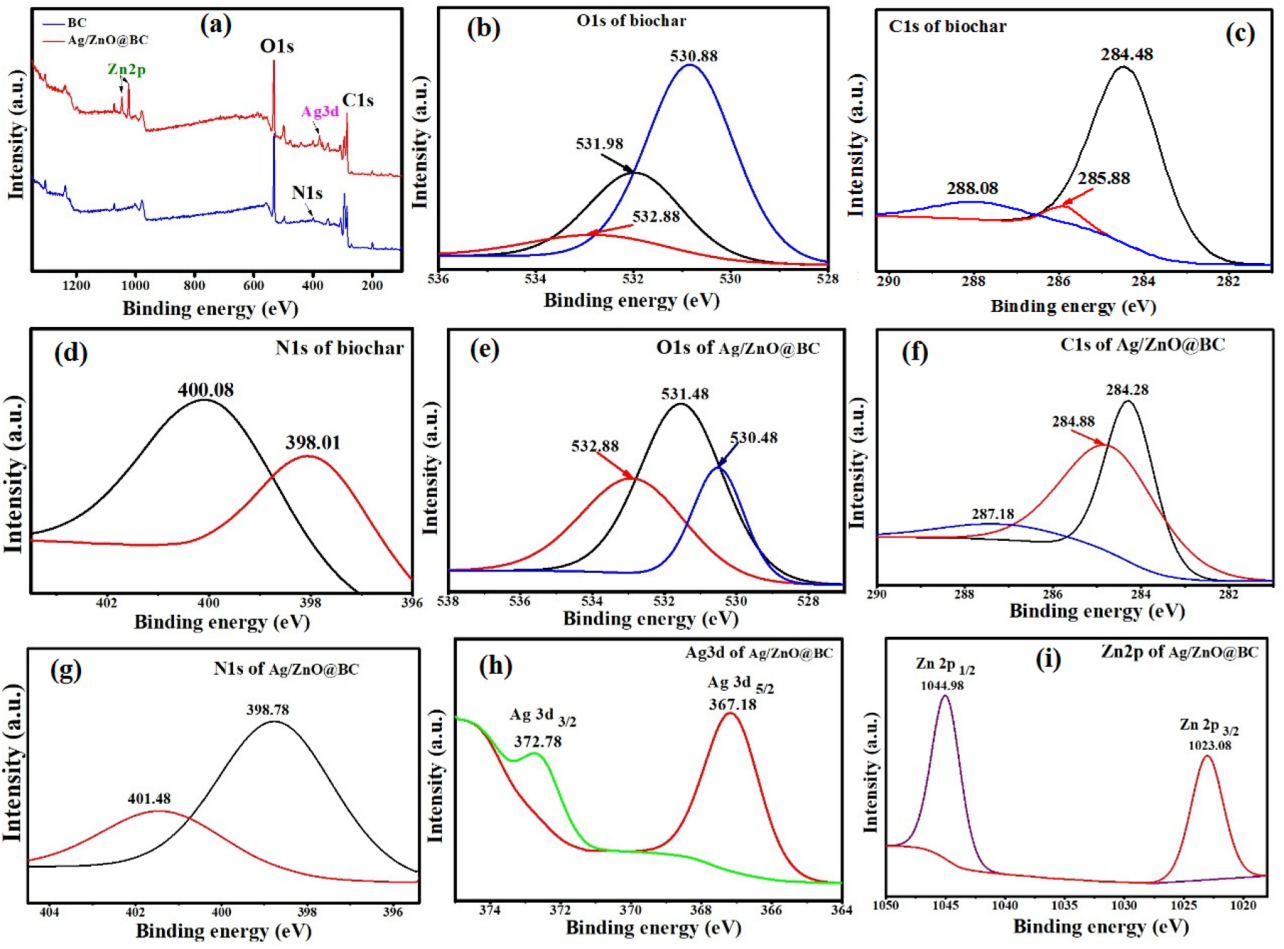
XPS spectra for biochar and Ag/ZnO@BC survey (**a**), biochar O1s (**b**), C1s (**c**), N1s (**d**), Ag/ZnO@BC O1s (**e**), C1s (**f**) N1s (**g**), Ag3d (**h**) and Zn2p (**i**).

The ionic properties and bonding configuration changes between biochar and Ag/ZnO@BC were further investigated using XPS which is considered a strong surface technique. Main surveys for biochar and Ag/ZnO@BC indicate the presence of C1s, O1s, and N1s as major constituents. In addition, Zn2p and Ag3d appeared in the case of Ag/ZnO@BC nanocomposite as shown in Fig. 7a. Figure 7c shows the C1s spectrum of the biochar and the different peaks at 284.48 eV, 285.88 eV, and 288.08 eV are attributed to C–C, C=C, and C–O, respectively, which are generally resulting from the polyphenol groups of the plant^53^. In comparison with the C1s of the Ag/ZnO@BC (Fig. 7f), there is an obvious shift in the C–O peak at 288.08 eV to 287.18 eV, and another one was observed in the C=C that shifted from 285.88 to 284.88 eV with a large intensity indicating the reduction of Ag ions into AgNPs and the formation of ZnONPs on the biochar surface. The binding energies of the O1s spectrum of the biochar (Fig. 7b) show that the binding energy peak at 530.88 eV, 531.98 eV, and 532.88 eV were attributed to the O atoms from sulphonate function^54^, S=O group^55^ and C–O group^56^, respectively. The O1s of the Ag/ZnO@BC (Fig. 7e) shows that there was an unequivocal change in the intensity of these peaks at 530.48 eV, 531.48 eV, and 532.88 eV denoting the Zn–O bonding as the intensity of these peaks partially associated with the changes in the oxygen vacancy concentration^57^ and the bounding of AgNPs to the biochar surface. Furthermore, the N1s spectrum of biochar showed the presence of C–N at 398 eV and 400.08 eV as presented in Fig. 7d that shifted to 398.78 eV and 401.48 eV in the case of Ag/ZnO@BC (Fig. 7g) signifying the formation of ZnO and AgNPs and their possible interaction with nitrogen^58^. The Ag3d spectrum (Fig. 7h) showed two peak binding energies at 367.18 eV and 372.78 eV corresponding to the unbound Ag3d_5/2_ and Ag3d_3/2_, respectively of AgNPs since the binding energy difference was nearly 6 eV similarly to Ravikumar et al.^51^ who synthesized a nanocomposite of Ag-TiO2@Pd/C for the sake of ofloxacin photodegradation. Figure 7i showed the peaks of Zn2p_3/2_ and Zn2p_1/2_ at 1023.08 and 1044.98 eV, respectively. It has to be mentioned that the binding energy difference of 21.9 eV between these two peaks confirmed the presence of zinc in the Zn^2+^ oxidation state as in line with Krishnakumar et al.^59^ who prepared a nanocomposite of AgBr–ZnO. Consequently, the XPS analysis indicated the occurrence of ZnO and AgNPs on the Ag/ZnO@BC surface.

### Thermal gravimetric analysis (TGA)

**Figure 8:**
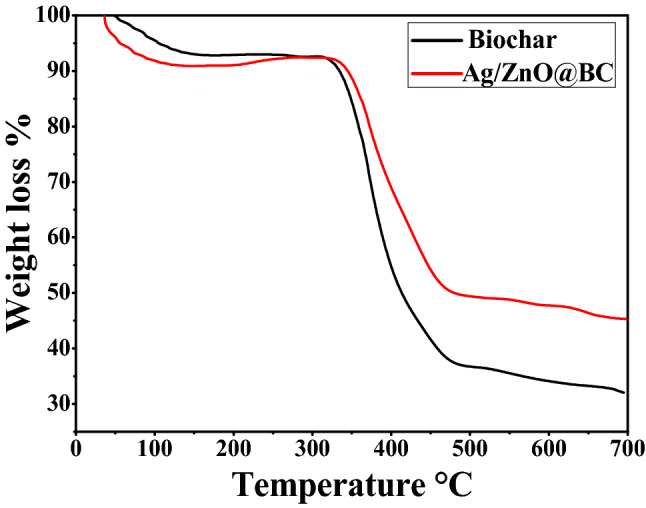
TGA curves of biochar and Ag/ZnO@BC.

TGA analysis of Ag/ZnO@BC and biochar are displayed in Fig. 8. Ag/ZnO@BC demonstrated a first systematic stage with near weight loss of 10% up to 140 °C while the biochar sample showed a weight loss of 7% nearly up to the same temperature that could be accredited to the moisture content loss. Further, the Ag/ZnO@BC and biochar were practically stable up to 330 °C and 320 °C. Subsequently, a significant weight loss from 330 to 475 °C for Ag/ZnO@BC and from 320 to 490 °C for biochar sample occurred that may be accredited to the breakdown of cellulosic and hemicellulosic compounds^60^. Eventually, there was a slight weight loss for both samples up to 700 °C that could be related to the decomposition of the lattice structure of both samples^61^ but with a higher degree of weight loss in the case of pristine biochar. The obtained result is in line with Inyang et al.^62^ who improved the thermal stability of biochar by modifying it with carbon nanotubes. Thus, it is obvious that the total weight loss of Ag/ZnO@BC was lower compared to the biochar that could be related to the zinc oxide and silver nanoparticles' capacity of resisting thermal decomposition.

### XRD analysis

**Figure 9:**
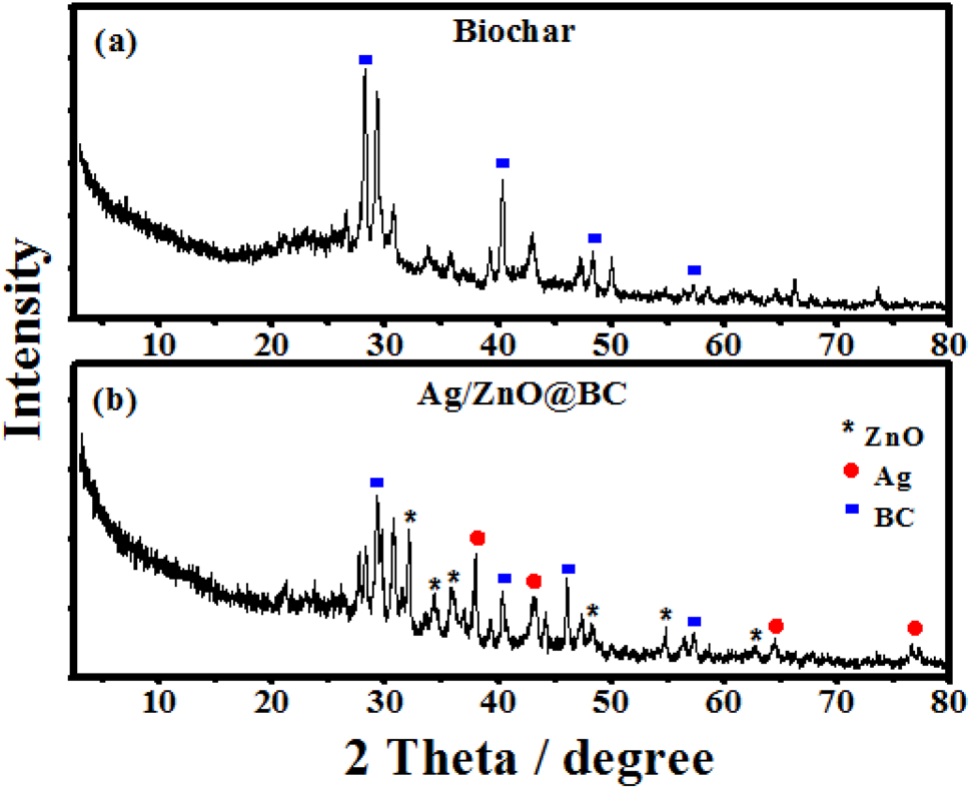
XRD patterns of biochar (**a**) and Ag/ZnO@BC (**b**).

XRD spectrum of the biochar (Fig. 9a) demonstrated distinguishing peaks at 28.19°, 40.37°, 48.53°, and 57.31° that were indexed to (002), (100), (101), and (004) planes, respectively, which were mentioned by other researchers^63^, while the XRD of Ag/ZnO@BC nanocomposite (Fig. 9b) revealed the same peaks of the biochar but with a lesser intensity and slightly shifted positions in addition to other new characteristic peaks of AgNPs were observed at 38°, 43.35°, 64.47° and 77.19° which are attributed to (111), (200), (220), and (311) planes referring to face-centered-cubic (FCC) silver [JCPDS file number 04-0783]. Moreover, the (111) plane, in line with numerous researchers, was the preferred growth direction for the phytosynthesized AgNPs on the surface of Ag/ZnO@BC nanocomposite. Furthermore, other peaks were detected at 32.15°, 34.35°, 36.03°, 48.49°, 54.77°, and 62.83°. These peaks are indexed as (100), (002), (101), (102), (110), and (103) and confirmed the presence of crystalline phytosynthesized ZnONPs with hexagonal wurtzite structure [JCPDS file number 36-1451]. These results were in agreement with Yu et al.^41^ who used the ball milling method for the synthesis of ZnO-biochar. Thus, the current results confirmed the successful phytosynthesis of both AgNPs and ZnONPs on the biochar surface resulting in the production of Ag/ZnO@BC nanocomposite.

### Photocatalytic degradation of TC

**Figure 10:**
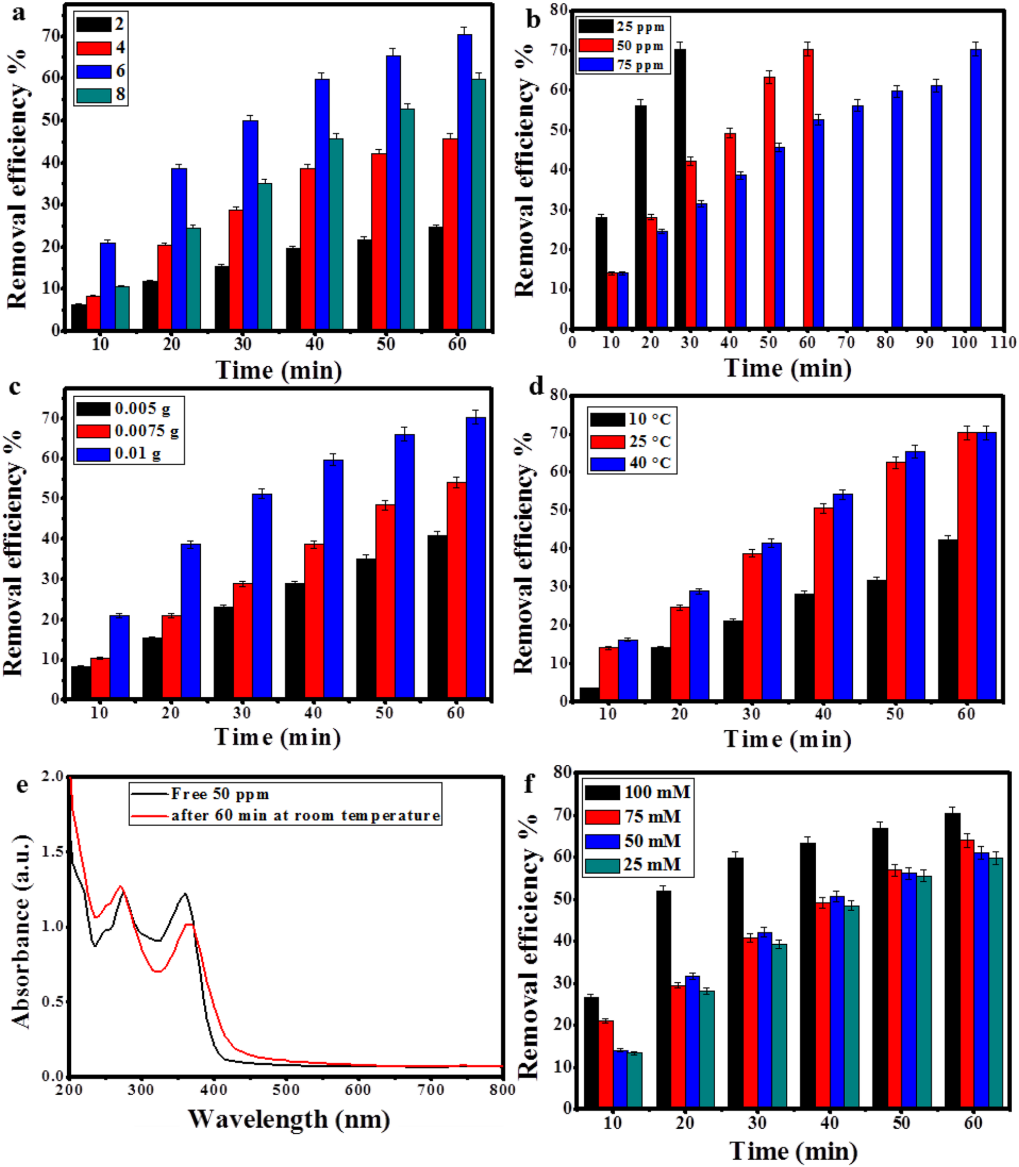

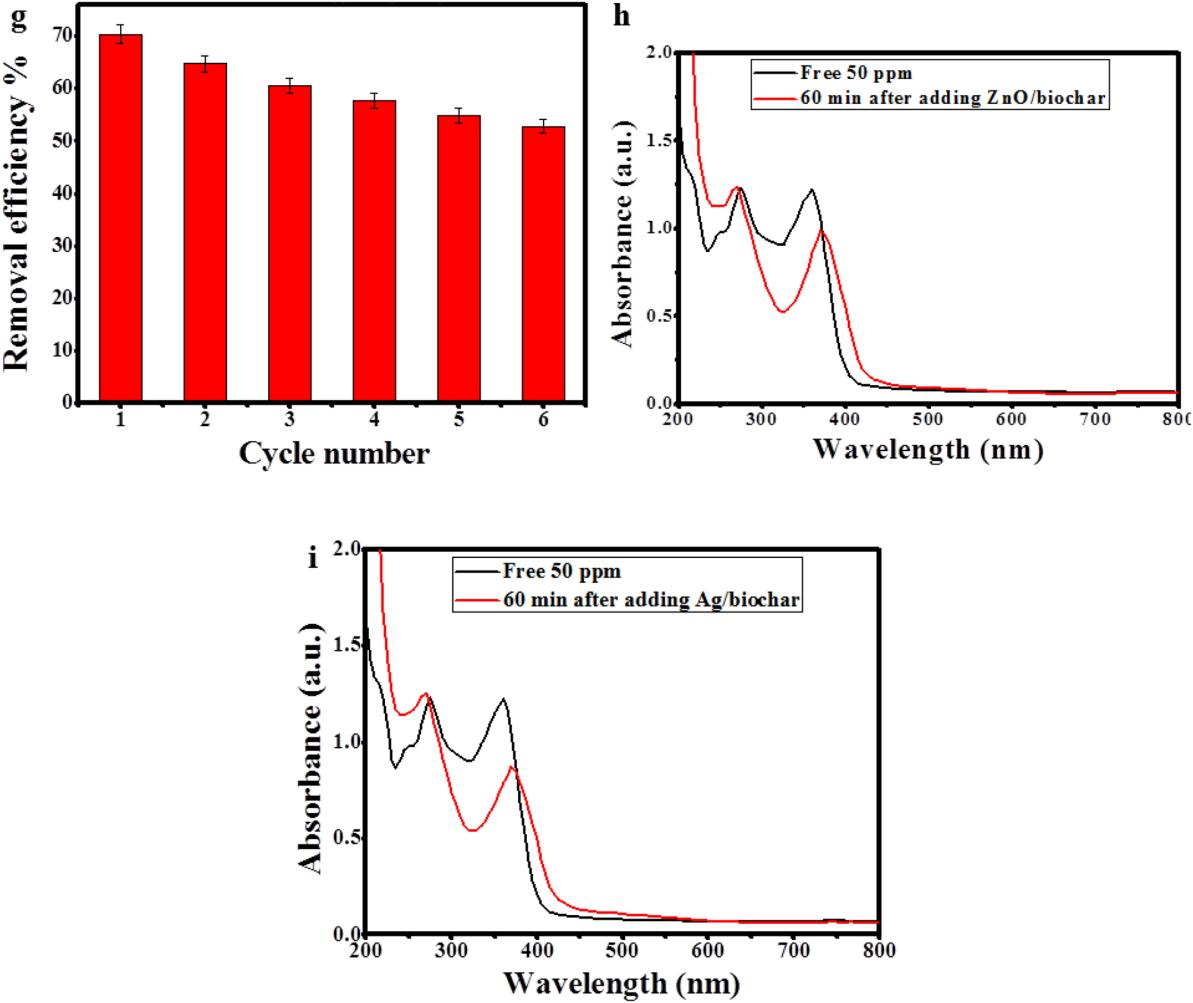
Effect of pH (**a**), initial TC concentration (**b**), Ag/ZnO@BC's dose (**c**), temperature on the removal efficiency of TC (**d**), Photocatalytic degradation of TC after 60 min using room temperature (**e**), effect of H_2_O_2_ concentration on the removal efficiency of TC (**f**), recyclability of Ag/ZnO@BC (**g**), Photocatalytic degradation of TC after 60 min using ZnO/biochar (**h**), and Ag/biochar (**i**).

In this study, several factors affecting the photodegradation process of TC were investigated including the pH level, initial concentration of TC, the dose of Ag/ZnO@BC, the temperature of the reaction, and the free radicals effect such as H_2_O_2_ to search for the optimum conditions for the removal of TC. The pH was firstly inspected within 1 h of photodegradation of 50 ppm TC as it is regarded to have the major effect on the photocatalytic degradation since the pH affects the surface charge and electron transfer ability of the photocatalyst^64^. When the reaction condition was highly acidic (pH 2), the removal efficiency was only 24.68%. Subsequently, the removal efficacy improved to 45.69% at pH 4, then it boosted to almost 70.3% at pH 6. However, it diminished to approximately 60% at the alkaline pH level (pH 8), which could be attributed to the decomposition of H_2_O_2_ into O_2_ instead of forming the OH radicals that are highly required for the photodegradation of TC^65^. Therefore, Ag/ZnO@BC was proved to be harnessed as a photocatalyst for TC photodegradation over a wide range of different pH levels (Fig. 10a).

Regarding the variation in the initial concentration of TC from 25 to 75 ppm, the photodegradation efficiency was observed to be approaching 70.3% with all the tested concentrations as shown in Fig. 10b yet in different times as it took 30, 60, and 100 min to reach 70.3% photodegradation efficiency for 25, 50, and 75 ppm under pH 6. Thus, indicating the high efficacy of Ag/ZnO@BC in the application of TC removal with various concentrations.

The effect of variation in Ag/ZnO@BC's dose on the removal efficiency of TC was conducted in the current work as presented in Fig. 10c within 1 h of photodegradation of 50 ppm TC under pH 6. Subsequently, it was concluded that with varying the catalyst dose from 0.005 to 0.0075 g and 0.01 g, the photodegradation efficiency escalated from 40.7 to 54.1 and 70.3%, respectively, which is in agreement with^66^ who reported a similar result.

Concerning the temperature effect on the TC removal with a concentration of 50 ppm in 1 h at pH 6 (Fig. 10d), it was detected that the efficiency was only 42.1% at the temperature of 10 °C. However, the efficiency boosted and reached almost 70.3% when the temperature increased to 25 °C. Additionally, the same degradation percentage was obtained when the temperature was raised to 40 °C yet with a slightly faster rate. Also, it has to be noticed that when the effect of temperature was tested in the absence of Ag/ZnO@BC, the degradation efficiency was only around 14% as shown in Fig. 10e indicating the minor influence of bare temperature on the TC degradation. It could be suggested that the photocatalytic degradation becomes quicker by raising temperature as a result of the increased production of hydroxyl free radicals. Thus, the intermediate temperature (25 °C) was utilized for the rest of the experiments to mimic the natural conditions of wastewater treatment plants.

In order to determine the H_2_O_2_ effect on the photodegradation process of TC, four different concentrations of H_2_O_2_ (25, 50, 75, and 100 mM) were experimented with the TC solutions (50 ppm) under pH 6 for 1 h as shown in Fig. 10f. In the lowest concentration, the achieved degradation percentage was nearly 59.75%. Subsequently, the degradation percentage increased to 61.1%, 63.9%, and 70.3% with increasing the concentration of H_2_O_2_ to 50, 75, and 100 mM. Thus, the effect of H_2_O_2_ addition was confirmed to enhance the removal of TC as it provides a source of hydroxyl groups, which resulted in supplying the photodegradation system with extra hydroxyl radicals^67^.

When the stability and recycling of Ag/ZnO@BC as a photocatalyst was inspected (Fig. 10g) within 1 h of photodegradation of 50 ppm TC at pH 6, it was indicated that the efficiency of Ag/ZnO@BC diminished from 70.3 to 53% after six cycles of utilization, denoting the well efficacy of Ag/ZnO@BC reuse. In addition, it has to be clarified that the biochar was used as a support for AgNPs and ZnONPs to facilitate the regeneration process of these nanoparticles and obtain high degradation efficiencies for TC as in line with Liu et al.^68^ who enhanced the degradation efficiency of TC from 67.3 to 92.5% by synthesizing a nanocomposite of Fe-Cu-biochar compared to Fe-Cu alone. Also, when the photocatalytic efficiency of ZnO/biochar and Ag/biochar was tested in degrading TC, it was found that the degradation efficiency was approximately 21% for ZnO/biochar and 29% for Ag/biochar as displayed in Fig. 10h, i, respectively, confirming the synergistic effect of AgNPs and ZnONPs together in enhancing the degradation efficiency of TC up to 70.3%.

**Figure 11:**
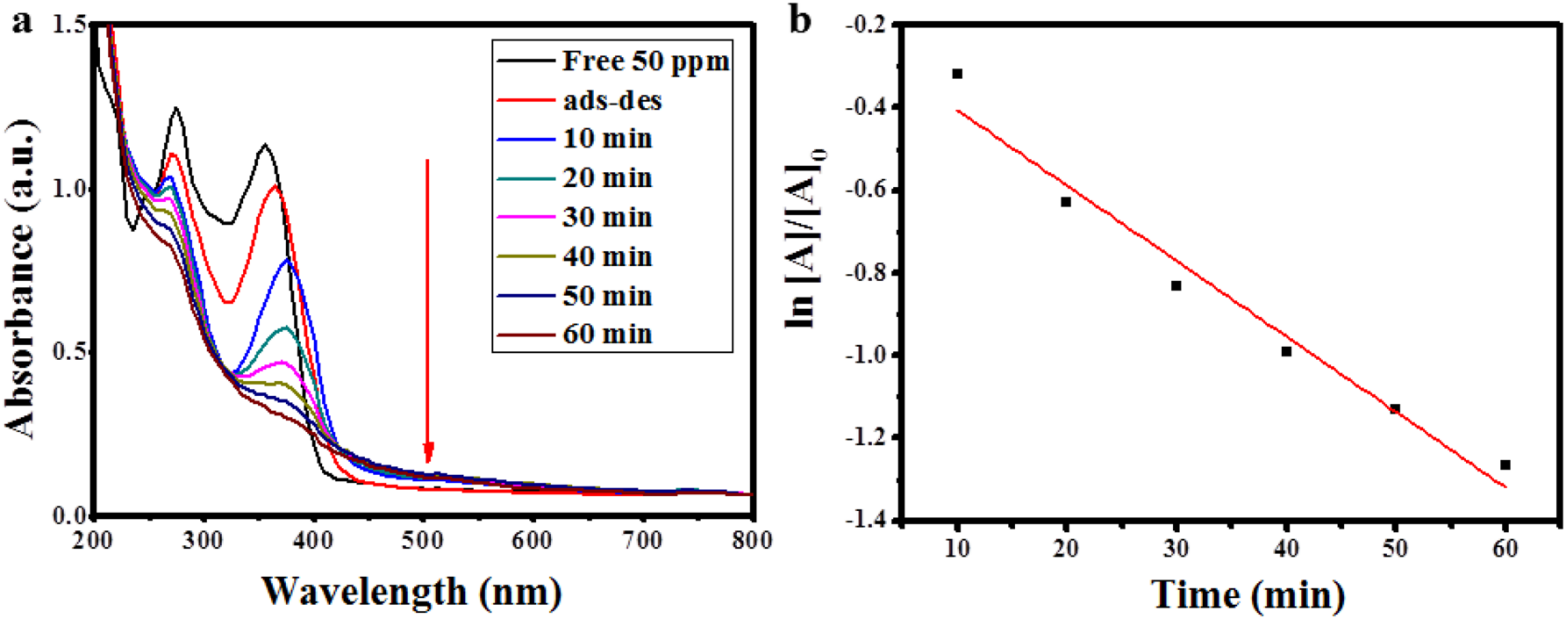
UV–Vis absorption spectrum for the photodegradation of TC by Ag/ZnO@BC under the optimum conditions (TC concentration 50 ppm, pH 6, dose of Ag/ZnO@BC 0.01 g, temperature of 25 °C, and H_2_O_2_ concentration of 100 mM) (**a**) Kinetics of photocatalytic degradation of TC under the optimum conditions (**b**).

According to the abovementioned results, the optimum conditions for the removal of TC were determined to be pH 6, the dose of Ag/ZnO@BC 0.01 g, the temperature of 25 °C, and H_2_O_2_ concentration of 100 mM. When these experimental conditions were carried out experimentally with an intermediate concentration of TC (50 ppm), the removal efficiency reached 70.3% in 60 min as shown in Fig. 11a that is similar to the degradation efficiency of 77% obtained by Shi et al.^69^ who applied green synthesized nanosheets of carbon-doped graphitic carbon nitride in 1 h. Kinetics of the photodegradation of TC under the optimum conditions are presented in Fig. 11b and the rate constant K was 0.0182 min^−1^. Therefore, when UV light interacted with Ag/ZnO@BC, electron–hole pairs were formulated owing to the SPR phenomenon of both ZnONPs and AgNPs resulting in the formation of reactive oxygen species (ROS) such as superoxide anion (^•^O_2_^−^) by the reaction of free electrons e^−^ with oxygen as well as hydroxyl radicals (^•^OH) through the reaction of h^+^ with H_2_O molecules on the surface of Ag/ZnO@BC, which in turn leads to the degradation of TC that is in line with the result obtained by Pan et al.^70^ who targeted the degradation of TC using nanodiamonds/UiO-66-NH_2_, Sun et al.^71^ who utilized Fe-doped g-C_3_N_4_, as well as Guo et al.^72^ who used carbon nitride decorated with Cu_3_P nanoparticles. Also, the photodegradation of TC could be enhanced by the action of other ROS species such as the singlet oxygen (^1^O_2_) that usually results from the reaction of UV light with oxygen (O_2_)^73^.

### Antibacterial study

The well-known inhibitory properties of Ag and ZnO nanoparticles have been used in a range of therapeutic applications^74^ most notably the inhibition of gram-positive and gram-negative bacterial strains. Subsequently, the antimicrobial efficacy of Ag/ZnO@BC synthesized in this work was tested against different gram-negative bacteria such as *Escherichia coli* and *Klebsiella pneumonia* and also gram-positive bacteria including *Bacillus subtilis* and *Staphyllococus aureus*. The bactericidal function of the impregnated nanoparticles is thought to be a process of two steps; firstly, they interact with thiol groups in proteins, causing inactivation, and secondly their interaction with bacterial DNA, condensing the DNA and preventing DNA replication leading to apoptosis^75^.

**Figure 12:**
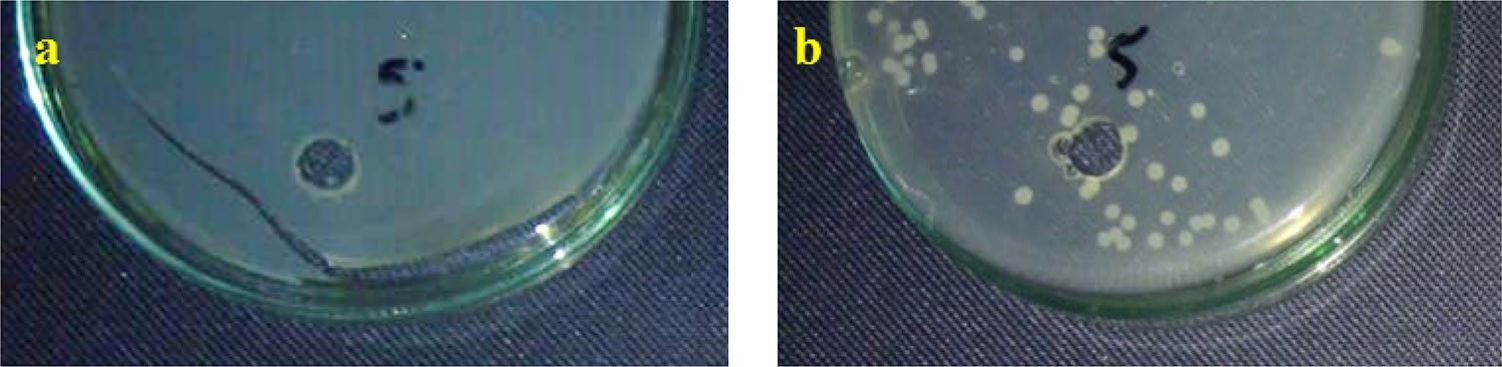
Antimicrobial effect of Ag/ZnO@BC against *Klebsiella pneumonia* (**a**) and *Escherichia coli* (**b**)*.*

The obtained results indicated that Ag/ZnO@BC is a strong antibacterial agent towards *Klebsiella pneumonia* as it prevented it’s growth at all (Fig. 12a) with a high concentration (2 × 10^8^ CFU/mL), particularly when compared with other green synthesized AgNPs, ZnONPs, and their nanocomposites that are presented in Table 1 such as Pd-RGO-ZnO that was fabricated by Rajeswari et al.^76^ and resulted in an inhibition zone of 11 mm against *Klebsiella pneumonia*. Also, our nanocomposite was better than Ag-ZnONPs that led to an inhibition zone of 24 mm against the same bacterial strain^27^. However, Ag/ZnO@BC could not stop the growth of *Escherichia coli* (Fig. 12b). Therefore, Ag/ZnO@BC can be utilized as an efficient antibacterial material in wastewater disinfection from *Klebsiella pneumonia*.

**Table 1:** Comparison between the antibacterial efficiency of Ag/ZnO@BC prepared in the current study and other AgNPs, ZnONPs, and nanocomposite prepared in other studies.

Sample	Sample concentration (μg mL^−1^)	Bacterial strain	Zone of inhibition (mm)	References
AgNPs	200	*Pseudomonas aeruginosa*	27	^77^
Ag-ZnO NPs	0.25	*Klebsiella pneumonia*	24	^27^
		*Escherichia coli*	18	
		*Bacillus subtilis*	16	
		*Staphylococcus aureus*	16	
Ag-doped ZnO nanoparticles	–	*Bacillus subtilis*	15.5	^78^
		*Staphylococcus aureus*	17	
Chitosan-AgNPs (CS-AgNPs)	1	*Escherichia coli*	10	^79^
ZnONPs	–	*Staphylococcus aureus*	7	^80^
		*Escherichia coli*	12	
ZnONPs-Cellulose nanocomposite		*Staphylococcus aureus*	14.5	
		*Escherichia coli*	11	
Ag/ZnO-NCs	10	*Escherichia coli*	No growth (resistant)	^81^
polypyrrole/zinc oxide/chitosan bionanocomposite	–	*Staphylococcus aureus*	28.63	^82^
		*Bacillus subtilis*	20.83	
		*Escherichia coli*	17.70	
		*Pseudomonas aeruginosa*	29.60	
Ag/GO nanocomposite	1	*Staphylococcus aureus*	15	^83^
		*Escherichia coli*	19	
Pd-RGO-ZnO nanocomposite	–	*Pseudomonas aeruginosa*	10	^76^
		*Klebsiella pneumonia*	11	
Ag-ZnO nanocomposite	–	*Staphylococcus aureus*	8.6	^84^
		*Escherichia coli*	9.3	
Ag/ZnO@BC	10	*Escherichia coli*	Resistant	The current study
		*Klebsiella pneumonia*	No growth (sensitive)	
		*Bacillus subtilis*	Resistant	
		*Staphyllococus aureus*	Resistant	

### Antioxidant test

**Figure 13:**
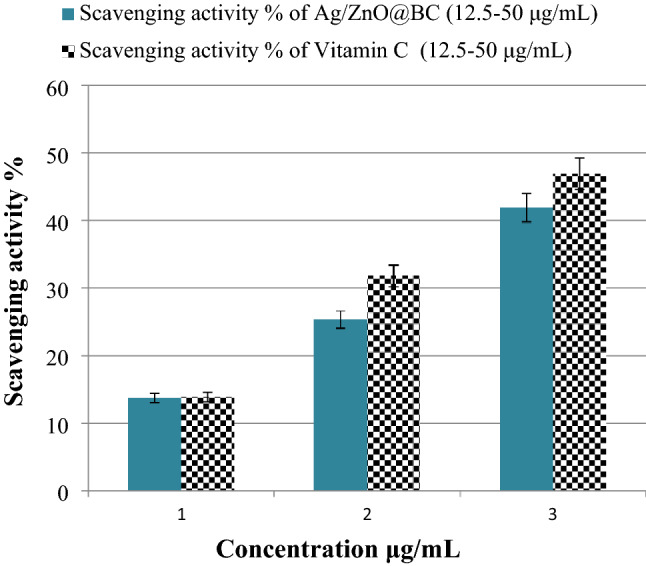
Antioxidant efficiency of Ag/ZnO@BC and Vitamin C (positive control) against DPPH.

Oxidative stress and other health problems are generally resulting from free radicals^85^. DPPH, which is a common toxic free radical, has been confirmed to cause adverse effects on human health. The antioxidant properties of Ag/ZnO@BC are probably resulting from the donation of electrons from the highly-dense oxygen atom of this nanocomposite to the nitrogen atom's odd electron in the DPPH molecule, resulting in the attenuated intensity of n → π* transition at 517 nm and the disappearance of DPPH characteristic violet color^86^. In this work, the scavenging% of DPPH improved exponentially from 13.77 to 41.89% by increasing the concentration of Ag/ZnO@BC increased from 12.5 to 50 µg/mL (Fig. 13) which is considered as a good percentage and concomitant with scavenging percentages reported by other workers including RGO-ZnO nanocomposite that achieved an antioxidant efficiency of 45%^76^ and better than ZnONPs-Cellulose nanocomposite that was synthesized by Ali et al.^80^ and attained only 14.85% of DPPH scavenging. Vitamin C attained 13.9%, 31.8%, and 46.9% of DPPH scavenging at the concentrations of 12.5, 25, and 50 µg/mL, in a respective manner, (Fig. 13) that are comparable with Ag/ZnO@BC. Consequently, the acceptable antioxidant potency of Ag/ZnO@BC was indicated against DPPH and its encouraging employment in the removal of other free radicals.

A comparison between Ag/ZnO@BC and other nanomaterials including different nanocomposites efficacy in DPPH scavenging is presented in Table 2.

**Table 2:** Comparison between the antioxidant efficiency of Ag/ZnO@BC prepared in the current study and other nanomaterials including various nanocomposites prepared in different studies.

Antioxidant	Concentration (µg/mL)	Scavenging activity	References
AgNPs	500	62	^87^
Chitosan-Ag nanocomposite	1000	80	^88^
ZnONPs	200	6.75	^80^
ZnONPs-Cellulose nanocomposite		14.85	
ZnONPs	500	75	^89^
Graphene		41	
Graphene-ZnONPs composite		81.83	
Reduced graphene oxide (RGO)	200	25	^76^
reduced graphene oxide/zinc oxide nanocomposite (RGO-ZnO)		45	
Palladium-Decorated reduced graphene oxide/zinc oxide nanocomposite (Pd-RGO-ZnO)		55	
Gelatin-Cellulose nanofiber-Zinc oxide-Selenium nanocomposite	50	55	^90^
Gelatin-Cellulose nanofiber- Selenium nanocomposite		50	
Gelatin-Cellulose nanofiber-Zinc oxide nanocomposite		35	
Carboxymethyl cellulose-ZnO nanocomposite	50	5	^91^
Carboxymethyl cellulose-curcumin-ZnO nanocomposite		30	
Ag/ZnO@BC	50	41.89	The current work

## Conclusions

In the current study, a sustainable, cost-effective, and the completely green procedure was employed for the production of Ag/ZnO@BC nanocomposite utilizing *P. salicifolia* biomass is being reported for the first time. The phytosynthesized nanoparticles on the Ag/ZnO@BC surface were mainly spherical and ranging from 20 to 30 nm. Numerous phytoconstituents in *P. salicifolia* biomass were suggested to be involved in the green synthesis of Ag/ZnO@BC including flavonoids, terpenoids, alkaloids, and glycosides. The stability of Ag/ZnO@BC was designated via a zeta potential value of − 24.6 mV. Ag/ZnO@BC displayed high efficiency in the photocatalytic degradation of TC that reached 70.3% under the optimum reaction conditions including TC concentration; 50 ppm, pH; 6, the dose of Ag/ZnO@BC; 0.01 g, temperature of 25 °C, and H_2_O_2_ concentration of 100 mM. Moreover, the reusability of Ag/ZnO@BC was acceptable as it reached 53% after the sixth cycle of reuse and the rate constant K was 0.0182 min^−1^. Moreover, Ag/ZnO@BC demonstrated a substantial antibacterial activity against *Klebsiella pneumonia* as well as a promising antioxidant activity with a maximum efficacy of 41.89% at the maximum concentration (50 µg/mL). Therefore, Ag/ZnO@BC constituted a novel nanocomposite that could be proficiently applied in a variety of environmental and medical uses.
